# Genome-wide association studies in non-anxiety individuals identified novel risk loci for depression

**DOI:** 10.1192/j.eurpsy.2022.32

**Published:** 2022-06-22

**Authors:** Bolun Cheng, Xin Qi, Peilin Meng, Shiqiang Cheng, Xuena Yang, Li Liu, Yao Yao, Yumeng Jia, Yan Wen, Feng Zhang

**Affiliations:** 1 Key Laboratory of Trace Elements and Endemic Diseases, Collaborative Innovation Center of Endemic Disease and Health Promotion for Silk Road Region, School of Public Health, Health Science Center, Xi’an Jiaotong University, Xi’an 710061, China; 2 Precision Medicine Center, The First Affiliated Hospital of Xi’an Jiaotong University, Xi’an, China

**Keywords:** Depression, genome-wide association study, linkage disequilibrium score regression, non-anxiety

## Abstract

**Background:**

Depression is a debilitating mental disorder that often coexists with anxiety. The genetic mechanisms of depression and anxiety have considerable overlap, and studying depression in non-anxiety samples could help to discover novel gene. We assess the genetic variation of depression in non-anxiety samples, using genome-wide association studies (GWAS) and linkage disequilibrium score regression (LDSC).

**Methods:**

The GWAS of depression score and self-reported depression were conducted using the UK Biobank samples, comprising 99,178 non-anxiety participants with anxiety score <5 and 86,503 non-anxiety participants without self-reported anxiety, respectively. Replication analysis was then performed using two large-scale GWAS summary data of depression from Psychiatric Genomics Consortium (PGC). LDSC was finally used to evaluate genetic correlations with 855 health-related traits based on the primary GWAS.

**Results:**

Two genome-wide significant loci for non-anxiety depression were identified: rs139702470 (*p* = 1.54 × 10^−8^, OR = 0.29) locate in *PIEZO2*, and rs6046722 (*p* = 2.52 × 10^−8^, OR = 1.09) locate in *CFAP61.* These associated genes were replicated in two GWAS of depression from PGC, such as rs1040582 (*p*_replication GWAS1_ = 0.02, *p*_replication GWAS2_ = 2.71 × 10^−3^) in *CFAP61*, and rs11661122 (*p*_replication GWAS1_ = 8.16 × 10^−3^, *p*_replication GWAS2_ = 8.08 × 10^−3^) in *PIEZO2.* LDSC identified 19 traits genetically associated with non-anxiety depression (*p* < 0.001), such as marital separation/divorce (*rg* = 0.45, SE = 0.15).

**Conclusions:**

Our findings provide novel clues for understanding of the complex genetic architecture of depression.

## Introduction

Depression is one of the complex and common neuropsychiatric disorders affecting approximately 4.4% of the population worldwide, which often presents with low self-esteem, low mood, anhedonia, feeling of worthlessness, fatigue, sense of rejection and guilt, suicidal thoughts, among others [1, 2]. The lifetime prevalence of major depression around the world is between 1.0 and 16.9% [3]. Scientists have identified a familial tendency for depression through two decade of family studies [4]. However, a study sponsored by the Psychiatric Genomics Consortium (PGC) in 2013 did not find any associated loci of genetic variation in depression [5], and a subsequent meta-analysis conducted by Hek et al. was similarly inconclusive [6]. Like other common diseases or traits, depression is thus presumed to be controlled by many genes with minor effects.

Due to the wide range of depression phenotypes, previous studies could not achieve the statistical power required to test these minor effect variations. Therefore, PGC again combined the cohort data from PGC, UK BioBank, FinnGen and 23andMe to find some genetic associations for depression [7]. In the latest study, Levey et al. performed genome-wide association study (GWAS) and meta-analysis using depression data from more than 1.2 million participants in multiple population cohorts, and identified 17 pathogenic genes by fine-mapping GWAS signals combined with transcriptome association analysis [8]. Nevertheless, the existing studies seem to be not account for the influence of comorbidities to the pathogenesis of depression, and lack of further analysis and support of accurate phenotypic cohort data. Despite the previous family studies have shown that about 40% of depression susceptibility is due to genetic effects, little is known about the specific genetic variants involved in depression [4].

There is evidence that some mental disorders are typically heritable and share common genetic components [9, 10]. Anxiety and depression have been demonstrated to be bidirectional risk factors for one another [11]. Previous studies also found considerable overlapped genetic factors between anxiety and depression [12, 13]. A recent genetic correlation analysis found approximately 80% genetic correlation between depression and generalized anxiety [14]. However, there is a considerable pathogenesis difference between depression and anxiety [15]. For example, the release of some peptides or hormones on the hypothalamic–pituitary–adrenal (HPA) axis is regulated differently in anxiety and depression [16]. Besides, there are gender differences between the two diseases. Gao et al. found that anxiety was one of the most serious problems among female college students, while depression was relatively more serious among male [17]. Therefore, it is reasonable to explore depression specific genetic risk factors without the influence of anxiety. Our aim is to explore depression in the non-anxiety samples, and to eliminate common overlapping risk genes for anxiety and depression.

In this study, four genome-wide association studies were conducted using two depression phenotypes in two non-anxiety cohorts from the UK Biobank, respectively. Then, two GWAS summary statistics from PGC were used to verify the genes corresponding to the candidate loci in our GWAS. Finally, linkage disequilibrium score regression (LDSC) was performed to analyze the genetic correlation between non-anxiety depression and 855 health-related traits.

## Methods

### Non-anxiety depression samples in the UK Biobank cohort

The phenotypic and genotypic data of this study were derived from UK Biobank health resource (Application 46478), which had recruited 502,656 participants aged between 40 and 69 years [18]. The present study accessed health-related records of each participant, including age, sex, tobacco and alcohol consumption, and Townsend deprivation index (TDI) from screenshot question or verbal interview within Assessment Center. Anxiety (UK Biobank data fields: 20421 and 20420) and depression (UK Biobank data fields: 20002, 20126 and 20544) were defined based on the general anxiety disorder (GAD-7) and Patient Health Questionnaire (PHQ-9), respectively [19]. We used self-reported and mental illness scores to define the phenotype of depression and anxiety, respectively. Ethical approval of UK Biobank was granted by the National Health Service National Research Ethics Service (reference 11/NW/0382). Anxiety and depression score were mean-centered and normalized to one standard deviation (SD) before further analysis. The detailed definitions of mental phenotypes are shown in Supplementary File S1. In this study, individuals with an anxiety score <5 or non-self-reported anxiety were defined as non-anxiety individuals. The samples with anxiety score <5 included 59,334 depression cases (mean ± SD age, 57.22 ± 7.46) and 19,805 controls (mean ± SD age, 55.77 ± 7.40), while the samples with non-self-reported anxiety included 56,603 depression cases (mean ± SD age, 57.21 ± 7.48) and 13,123controls (mean ± SD age, 55.90 ± 7.45). The descriptive characteristics of participants with anxiety score <5 and non-self-reported anxiety are presented in Supplementary Files S2 and S3, respectively.

### UK Biobank genotyping, imputation, and quality control

In the UK Biobank, 488,377 participants have genome-wide genotype data. Genome-wide genotyping was conducted using either the Affymetrix UK BiLEVE Axiom or Affymetrix UK Biobank Axiom array. Details of the array design, genotyping, and quality control procedures have been descripted in the published study [18]. Imputation was conducted by IMPUTE2 against the reference panel of the Haplotype Reference Consortium, 1,000 Genomes and UK10K projects [18]. Detailed information about these data have been described elsewhere [20]. The SNPs with high linkage disequilibrium (*r*^2^ > 0.5) were removed. The participants were restricted to only “White British” according to self-reported ethnicity. The participants who reported inconsistencies between self-reported gender or genetic gender, and were genotyped but not imputed were finally excluded in this study.

### Genome-wide association studies of non-anxiety depression

PLINK 2.0 was used to conduct the GWAS of two depression traits in two non-anxiety cohorts, respectively [21]. For quality control, we removed the SNPs with call rates <90%, Hardy–Weinberg equilibrium (HWE) <0.001, or minor allele frequencies (MAF) <0.01. The kinship coefficients were estimated by KING software (http://people.virginia.edu/~wc9c/KING/) to remove the genetically related subjects [18]. The GWAS of depression score and self-reported depression were conducted in two non-anxiety cohorts using linear regression and logistic regression assuming an additive model for allelic effects, respectively. The age, sex, TDI, alcohol use frequency/week, smoking frequency/day, and top three principle components of population structure (calculated by UK Biobank) were used as covariates. The SNPs with *p* < 5.0 × 10^−8^ were considered to be genome-wide susceptibility significance.

### Replication of primary GWAS results

Two large-scale GWAS summary data of depression from the PGC were recruited to verify the accuracy of non-anxiety depression GWAS [7, 22]. Briefly, in replication GWAS 1, a genome-wide association meta-analysis was conducted based on 135,458 depression cases and 344,901 controls using logistic regression [7]. In replication GWAS 2, 807,553 discovery individuals (246,363 depression cases and 561,190 controls) were analyzed using logistic regression [22]. Detailed descriptions of genotyping, quality control and statistical analysis of these two data sets are available in the published studies [7, 22].

### Generating genetic correlations between non-anxiety depression and human traits

We used LDSC to estimate *rg* for non-anxiety depression with a range of other diseases and health-related traits [23]. The purpose of these comparisons was to assess the extent of shared common genetic variants in order to suggest hypotheses about the underlying genetic basis of non-anxiety depression. The overlap of the subjects themselves does not bias *rg.* These *rg* are mostly based on independent subject studies and are expected to be unbiased by confounding of genetic and nongenetic effects. *rg* remains unbiased when GWAS include overlapping samples, but the intercept of LDSC regression is an estimated correlation between association statistics attributable to overlapping samples. In this study, we used the cross-trait LDSC method through the LD Hub v1.9.3 to identify the genetic correlations between non-anxiety depression phenotype and 855 human diseases/traits, including physical and mental diseases, anthropometric markers, living habits and other health-related traits [24].

## Results

### Primary analysis of non-anxiety depression

**Figure 1.**
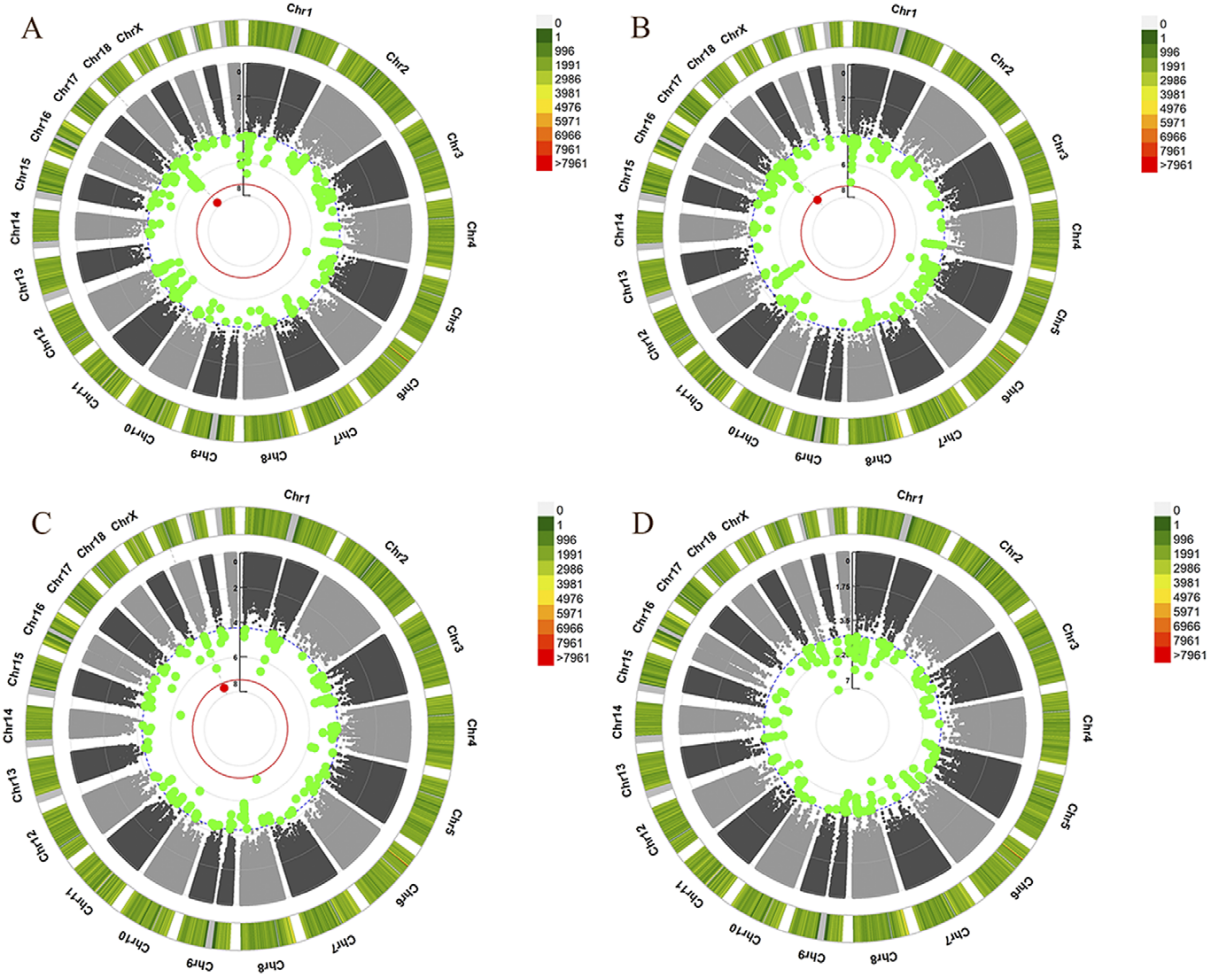
Manhattan plot for the GWAS of depression without anxiety in the UK Biobank cohorts. (A) Linear regression of depression score in anxiety score <5 samples. (B) Linear regression of depression score in non-self-reported anxiety samples. (C) Logistic regression of self-reported depression in anxiety score <5 samples. (D) Logistic regression of self-reported depression in non-self-reported anxiety samples. The red line indicates the *p*-value threshold for genome-wide significance (*p* < 5 × 10^−8^).

**Figure 2.**
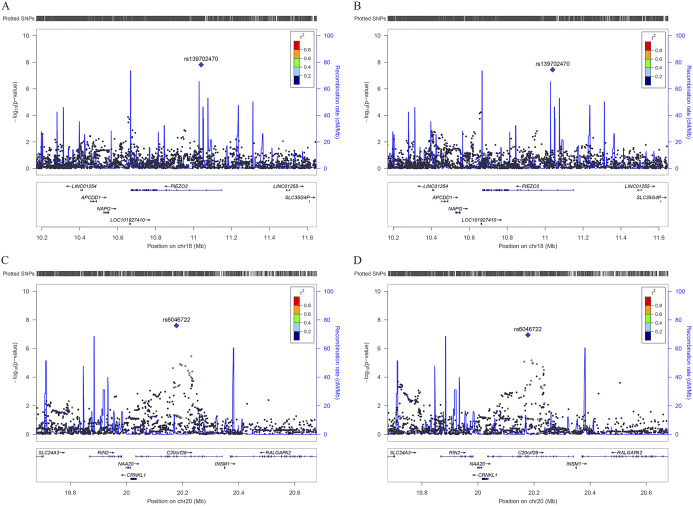
LocusZoom plots of depression without anxiety genome-wide significance loci. Association results for SNPs as a function of genomic distance for *PIEZO2* and *CFAP61* (*C20orf26*). The top line in each subfigure shows genomic coverage at the locus, with each vertical tick representing the imputed SNPs. Purple diamond indicate SNP at the locus with the strongest association evidence. Each point represents a SNP. Bottom panel shows genes at each locus as annotated in the UCSC Genome Browser Annotation Database. (A) display *PIEZO2* in chr18 for GWAS summary of depression score in anxiety score <5 samples. (B) display *PIEZO2* in chr18 for GWAS summary of depression score in non-self-reported anxiety samples. (C) display *CFAP61* in chr20 for GWAS summary of self-reported depression in anxiety score <5 samples. (D) display *CFAP61* in chr20 for GWAS summary of self-reported depression in non-self-reported anxiety samples.

An overview for the GWAS of non-anxiety depression is shown in Figure 1. In the analysis of self-reported depression in anxiety score <5 samples, one SNP reached genome-wide significance: rs6046722, *p* = 2.52 × 10^−8^, OR = 1.09 (Supplementary File S4). This SNP is located in an exon of the *CFAP61* (cilia and flagella associated protein 61) gene. In the GWAS of depression score in anxiety score <5 samples, rs139702470 reached genome-wide significance: *p* = 1.54 × 10^−8^, OR = 0.29 (Supplementary File S4). This is an intronic variant in the *PIEZO2* (piezo type mechanosensitive ion channel component 2) gene. In the analysis of depression score in non-self-reported anxiety samples, one SNP reached genome-wide significance: rs139702470, *p* = 3.66 × 10^−8^, OR = 0.29 (Supplementary File S4). This SNP is located in an exon of the *PIEZO2* gene. Figure 2 shows the LocusZoom plot with data coming directly from our GWAS summary data by querying the corresponding region on chromosomes between 500 and 500 kb region, respectively.

### Replication of primary analysis results

The genes corresponding to genome-wide significant loci for non-anxiety depression in the primary analysis were tested in two depression cohorts from the PGC. Both *CFAP61* and *PIEZO2* were associated with depression in the two replication studies. We observed 9 and 14 candidate SNPs corresponding to *CFAP61* and *PIEZO2* in two replication datasets (*p* < 0.05), respectively. For example, rs1040582 (*p*_replication GWAS1_ = 0.02, *p*_replication GWAS2_ = 2.71 × 10^−3^) and rs13038510 (*p*_replication GWAS1_ = 0.02, *p*_replication GWAS2_ = 9.83 × 10^−3^) were replicated in *CFAP61* region (Supplementary File S5), while rs11661122 (*p*_replication GWAS1_ = 8.16 × 10^−3^, *p*_replication GWAS2_ = 8.08 × 10^−3^) and rs11664237 (*p*_replication GWAS1_ = 8.58 × 10^−4^, *p*_replication GWAS2_ = 3.46 × 10^−3^) were replicated in *PIEZO2* region (Supplementary File S6).

### Linkage disequilibrium score regression

**Figure 3.**
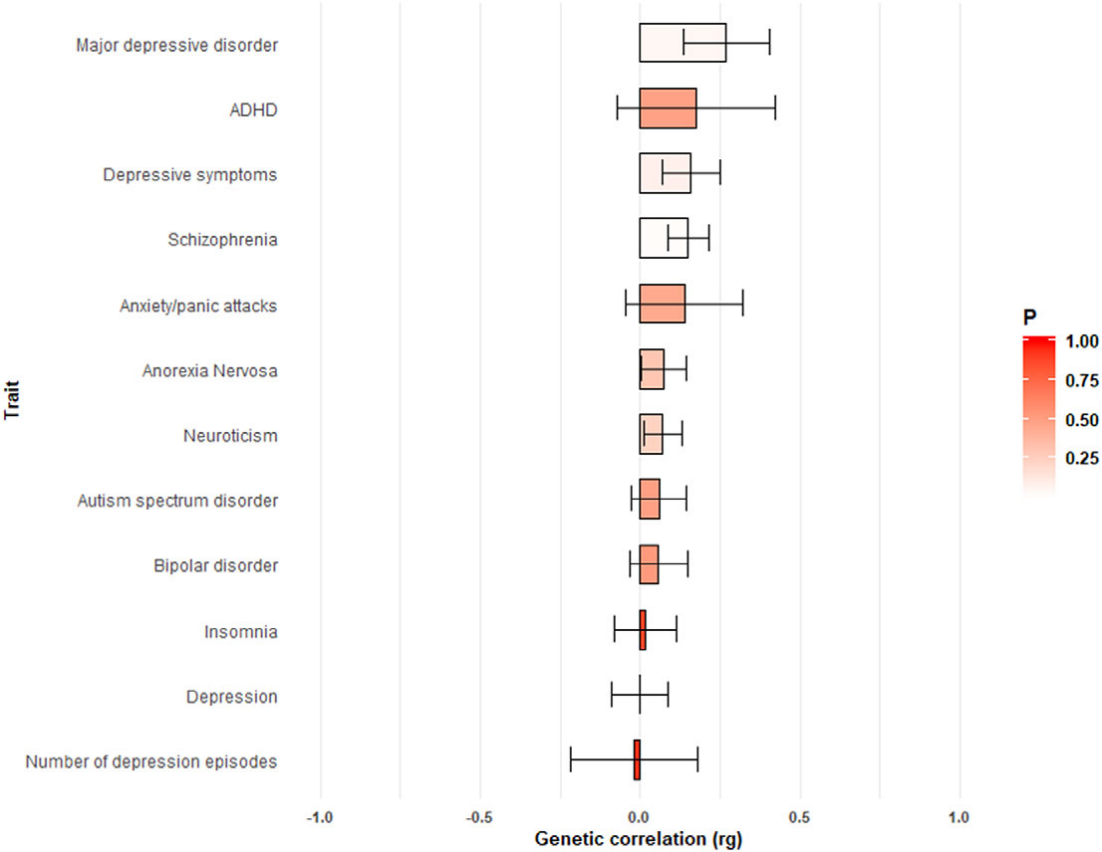
Genetic correlations and mental disorders related traits using LD score regression implemented in LD Hub software. The negative *rg* indicates that an earlier or lower value of a continuous trait was associated with depression without anxiety. The positive *rg* indicates that a later or higher value of a continuous trait was associated with depression without anxiety.

Non-anxiety depression is comorbid with a wide range of other diseases and disorders. To assess the shared genetic architecture between non-anxiety depression and many other traits, genetic correlations (*rg*) were calculated between our GWAS summary statistics and 855 behavioral and disease traits available via LD Hub. We first focused on the genetic correlations between non-anxiety depression and 12 common mental disorders (Figure 3). Suggestive significant genetic associations were detected for non-anxiety depression with major depressive disorder (*rg* = 0.27, SE = 0.13) and schizophrenia (*rg* = 0.15, SE = 0.06). Besides, several weak genetic correlations were observed between non-anxiety depression and many depression-related phenotypes, such as depression (*rg* = −0.001, SE = 0.09) and number of depression episodes (*rg* = −0.02, SE = 0.20). Notably, there was no significant genetic correlations between non-anxiety depression and other psychiatric phenotypes such as anxiety (*rg* = 0.14, SE = 0.18).

**Figure 4.**
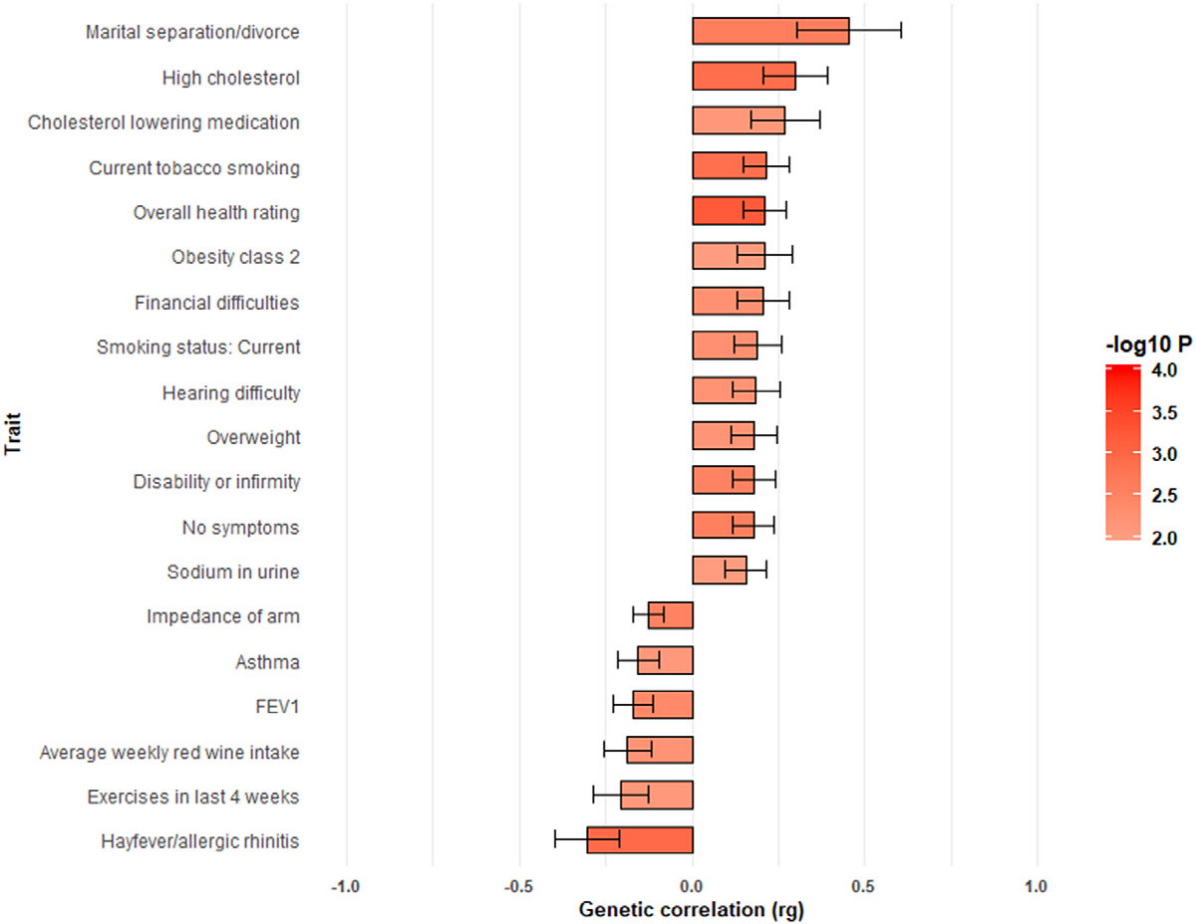
Significant genetic correlations and other behavioral and disease related traits using LD score regression implemented in LD Hub software. The negative *rg* indicates that an earlier or lower value of a continuous trait was associated with depression without anxiety. The positive *rg* indicates that a later or higher value of a continuous trait was associated with depression without anxiety.

Of the other behavioral and disease traits, 19 phenotypes were significantly correlated (*p* < 0.001) with non-anxiety depression (Figure 4), such as overall health rating (*rg* = 0.21, SE = 0.06), hayfever/allergic rhinitis (*rg* = −0.30, SE = 0.09), high cholesterol (*rg* = 0.30, SE = 0.09), current tobacco smoking (*rg* = 0.21, SE = 0.07), disability or infirmity (*rg* = 0.18, SE = 0.06), impedance of arm (*rg* = −0.13, SE = 0.04), and overweight (*rg* = 0.18, SE = 0.07). Additionally, a novel genetic correlation was observed between non-anxiety depression and marital separation/divorce (*rg* = 0.45, SE = 0.15) (Figure 4). Detailed results for genetic correlations between non-anxiety depression and other behavioral and disease related traits are summarized in Supplementary File S7.

## Discussion

In this study, we selected approximately 100,000 non-anxiety individuals from the UK Biobank, and conducted GWASs for depression scores and self-reported depression using the two non-anxiety cohorts, respectively. Our GWASs identified two independent SNPs associated with non-anxiety depression. Both of the genes corresponding to the two SNPs were verified by significant association signals across the two replication studies.

The genetic correlations between non-anxiety depression and the general depression phenotype were relatively weak in our study. A recent study using the UK Biobank cohort has found significant overlapping variants between depression and other mental disorders [25]. In contrast, our LDSC results did not find any strong genetic correlation between non-anxiety depression with neuroticism, anorexia nervosa, and anxiety/panic attacks. This supports our hypothesis that the analysis of non-anxiety depression may exclude the effects of comorbidities such as anxiety disorders. Interestingly, a significant genetic correlation was observed between schizophrenia and non-anxiety depression, indicating that schizophrenia may have a strong similarity to the genetic pathogenesis of depression.

Previous studies found that generalized anxiety and depression had a substantial genetic overlap, approximately 80–96% [14, 26]. Our study aimed to explore the depression specific genetic factors by excluding all anxiety individuals from the depression samples. In our LDSC analysis, the GWAS summary data of non-anxiety depression was generated from samples excluded anxiety individuals. On the contrary, the GWAS summary data of depression and number of depression episodes in LD Hub were not excluded anxiety individuals [24]. In this case, the *rg* correlation values between non-anxiety depression and depression traits (such as depression [−0.001] and number of depression episodes [−0.02]) were lower than that with anxiety [0.14], illustrating the potentially strong correlation between depression and anxiety. Thus, after the exclusion of the effect of anxiety individuals, we hypothesized that the genetic correlation was inevitably reduced between non-anxiety depression and depressive traits (including anxiety samples).

Examining significant genes that overlap between the current GWAS of non-anxiety depression and the depression studies revealed putative associations with *PIEZO2* and *CFAP61* [5, 7, 22]. *PIEZO2*, as the main biological force conduction medium, affects the release of neurotransmitter serotonin (5-HT) [27]. The 5-HT plays a central role in brain development, mood regulation, stress response, and the risk of psychiatric disorders, and changes of 5-HT have important implications for behavior and mental health [28]. *PIEZO2* is also associated with sensory nerve distribution in the central nervous system of the brainstem and cationic channels activated by brain metastatic cells [29, 30]. *PIEZO2* usually expressed in the cortical and hippocampal pyramidal neurons of the brain and in cerebellar Purkinje cells [31]. A whole-exome sequencing analysis in two patients who had unique neuromuscular and skeletal symptoms showed that *PIEZO2* was a determinant of mechanosensation in humans [32]. Lanier et al. conducted a study of brain injury caused by repeated blast exposure, and indicated that blast may cause *PIEZO2* change in sensitivity to mechanical stimuli in the brain and may contribute to cellular injury [33].

In our study, *CFAP61* was found to be significantly associated with non-anxiety depression and had been identified to play a vital role in primary cilia affecting cerebral cortical development and dysfunction [34]. It was highly expressed in preamygdala, striatum, and hippocampal structures [35]. However, the role of *CFAP61* in depression has not been well studied, and the role of *CFAP61* in the brain or nervous system is also limited. *CFAP61* gene identified by our GWAS is a remarkable finding for non-anxiety depression with respect to known biology and points to the potential value of other novel findings from this kind of research. Improving our understanding of the SNPs in non-anxiety depression may help identify the biological differences between depression and anxiety. These results also provide potential evidence for future phenotypic stratification research.

To date, neither *PIEZO2* nor *CFAP61* has been reported to cause a phenotype related to depression. It is important to note that the closest gene to the GWAS top signal is usually not the causal gene [36]. Besides, studies have shown that many genetic variants can affect phenotype through distal regulation such as long range enhancer–promoter interactions [37], and looping chromatin interactions [38]. Therefore, the SNPs identified in this study may affect the expression of other depression-related genes nearby. Several genes that are nearly adjacent to *PIEZO2* and *CFAP61* have been implicated in depression. For example, Dóra et al. carried out whole-exome ultra-high throughput sequencing in brain samples between depression and control subjects, and identified *GNAL* (near *PIEZO2*) as one of the genomic region-dependent accumulation of rare variants in depression [39]. Moreover, a polymorphism study in the alpha subunit found that *GNAL* gene was associated with major depression [40]. As a potassium-dependent Na^+^/Ca^2+^ exchanger, *NCKX3* (near *CFAP61*) is mostly abundant in the brain [41]. Behavioral examination in *NCKX3* knock-out mice found that depression-related behaviors in Nckx^−/−^ mice were more higher than that in wild type mice [41]. Thus, combined with our findings, the SNPs involved in this study may affect the pathogenesis of depression through regulating these adjacent genes.

The potential links between depression and obesity or smoking have been well speculated and repeatedly examined [42, 43]. Depression and obesity are common diseases with major public health implications, often cooccurring within individuals [44]. The current study also demonstrated that there existed significant genetic correlation between non-anxiety depression and obesity-related traits, including overweight, obesity class 2 and impedance of arm. Obesity can be seen as an inflammatory state because weight gain has been shown to activate inflammatory pathways, which in turn has been linked to depression [45–47]. In addition, smoking has been found to phenotypically and genetically correlate with depression [43, 48]. The current study also identified significant genetic correlations between non-anxiety depression and smoking-related traits, including current tobacco smoking and current smoking status. There have been many studies using different methods to demonstrate the complex causal relationship between depression and smoking, such as smoking increasing the risk of depression [49], a bidirectional effect [50] and no effect reported [51]. Our results largely support the previous association between depression and obesity or smoking.

Many epidemiological studies convey the same message: divorce threatens mental and physical health [52, 53]. There was a high genetic correlation between non-anxiety depression and marital separation/divorce (*rg* = 0.45) in our LDSC. It could be valuable to assess depressive phenotypes from a large cohort associated with access to marital status records. Separated and divorced people have a higher risk of mental illness than married people [54]. An earlier cross-sectional study focused on immune changes and found that separated/divorced men had poorer immune function and more recent illnesses [55]. In fact, the norepinephrine data for newlyweds matched the evidence linking divorce with increased inflammation [56]. In addition, those who had symptoms of depression before their divorce were more likely to develop depression after their divorce [57]. Future research should focus on how marriage and divorce can provoke health-relevant immune alterations, especially on immune and hormone-related non-anxiety depression.

Overall health rating is a common self-assessment score in epidemiological studies, and has been widely used as a powerful indicator in public health studies [58–60]. We observed moderate genetic correlation between non-anxiety depression and overall health rating (*rg* = 0.21), which was consistent with a similar research assessing the relationship between overall health rating score and major depressive disorder [61]. This genetic correlation for overall health rating may offer important new insights into the interrelationship between self-assessment health and depression. Future studies need to assess the pleiotropic confounding factors that could further explain genetic correlation between non-anxiety depression and overall health rating. In these studies, phenotypes should ideally be investigated without composite compositions and based on multiple phenotype indicators.

The principal strength of this study is to conduct a more accurate study on the premise of excluding the influence of anxiety comorbidities of depression in two large cohorts. This has allowed the validation of the effects of variants that have been identified previously to determine whether they maintain an effect on depression. We also recognize limitations in our study. In contrast to Europeans, other ancestors are still less well studied. We hope that the initial results of the UK sample reported here can help to advance the field by encouraging more collaborative research on other ethnic groups. Besides, there are some non-UK ethnic samples in LD Hub, which may have a slight bias on the accuracy of genetic correlation analysis, and future studies could further refine the phenotype.

## Conclusion

In summary, we identified two novel loci that should prioritize their further study in the pathology of non-anxiety depression. We examined genetic correlations between non-anxiety depression and 855 health-related phenotypes, largely confirming and strengthening previous observations. This study sheds light on the genetic architecture of depression and provides novel insights for future research of complex comorbid psychiatric traits.

## Data Availability

The data that support the findings of this study are available from the authors.

## Supplementary material

For supplementary material accompanying this paper visit http://doi.org/10.1192/j.eurpsy.2022.32. click here to view supplementary material
