# Supplementary material for: Genome-wide association studies in non-anxiety individuals identified novel risk loci for depression
Source: Eur Psychiatry. 2022 Jun 22;65(1):e38. doi: 10.1192/j.eurpsy.2022.32 (PMC9353885; doi:10.1192/j.eurpsy.2022.32)
Supplement: Supplementary file 1 [file S0924933822000323.zip › S0924933822000323sup001.docx]

**Genome-wide association studies in non-anxiety individuals identified novel risk loci for depression**

**Supplementary Material List**

Supplementary file 1. Definitions of criterion for phenotypes in UK Biobank cohort

Supplementary file 2. Descriptive characteristics of anxiety score < 5 participants

Supplementary file 3. Descriptive characteristics of non-self-reported anxiety participants

Supplementary file 4. Genome-wide significant loci for depression without anxiety showing the candidate genes

Supplementary file 5. Replication of primary analysis results in *CFAP61* region

Supplementary file 6. Replication of primary analysis results in *PIEZO2* region

Supplementary file 7. Genetic correlations between depression without anxiety and other behavioral and disease related traits using LD score regression implemented in LD Hub software
