# Supplementary material for: Genome-wide association studies in non-anxiety individuals identified novel risk loci for depression
Source: Eur Psychiatry. 2022 Jun 22;65(1):e38. doi: 10.1192/j.eurpsy.2022.32 (PMC9353885; doi:10.1192/j.eurpsy.2022.32)
Supplement: Supplementary file 1 [file S0924933822000323.zip › S0924933822000323sup002.docx]

**Supplementary file 1. Definitions of criterion for phenotypes in UK Biobank cohort**

***Definitions of criterion for anxiety score phenotype***

The 7-item anxiety scale (GAD-7) is a valid and efficient tool for screening for generalized anxiety disorder and assessing its severity in clinical practice and research[[1](#_ENREF_1)]. The core symptoms of anxiety were ID 20421 and 20420 according to CIDI-SF. Anxiety phenotype was defined according to seven UK biobank fields: 20505, 20506, 20509, 20520, 20515, 20516 and 20512. The anxiety participants were selected based on the ID 20421 and 20420. Excluding those that screen positive for mild anxiety means that there is greater confidence that this group have not had anxiety[[2](#_ENREF_2)].

***Definitions of criterion for self-reported anxiety phenotype***

The case group criteria of anxiety were defined self-reported according to the UK Biobank fields: 20002. Anxiety was selected based on the code 1287 from ID 20002, and code 15 from ID 20544 as case. In order to obtain a comprehensive and accurate control group, we strictly conducted the control group threshold by Davis et al. research[[2](#_ENREF_2)], which based on general anxiety disorder (GAD-7)[[3](#_ENREF_3)] and another strict criterion based on composite international diagnostic interview short-form (CIDI-SF)[[3](#_ENREF_3), [4](#_ENREF_4)].

For the control of anxiety, after excluding the anxiety defined in our study and generalized anxiety disorder (GAD) ever defined in Davis et al. research[[2](#_ENREF_2)], we chose the participants who did not endorse anxiety or screen positive on GAD-7. More precisely, participants whose GAD score <5. GAD-7[[3](#_ENREF_3)] is a classification algorithm with a total score (0-21) used to screen for and measure anxiety severity, focusing on seven anxious symptoms and signs (as detailed below：Feeling nervous, anxious or on edge 20506, Not being able to stop or control worrying 20509, Worrying too much about different things 20520, Trouble relaxing 20515, Being so restless that it is hard to sit still 20516, Becoming easily annoyed or irritable 20505, Feeling afraid as if something awful might happen 20512. In order to meet the 0-3 score for each item of GAD, the 7 symptom scores (1-4) of our team UK were all reduced by 1 point, which was then added up and participants with GAD score <5 were selected.

***Definitions of criterion for depression score phenotype***

The patient health questionnaire (PHQ-9) and composite international diagnostic interview short-form (CIDI-SF) were used to define the comprehensive and accurate dataset of depression[[3](#_ENREF_3), [4](#_ENREF_4)]. The depression group was selected based on depression phenotype, which was defined according to the coding 1286 from Data-Field 20002, coding 3, 4 or 5 from Data-Field 20126 and coding 11 from Data-Field 20544, excluding the self-reported depression and depression single episode defined in Davis et al.[[2](#_ENREF_2)].

***Definitions of criterion for self-reported depression phenotype***

The case group criteria of depression were defined self-reported according to three UK Biobank fields: 20544. Depression was selected based on the code 1286 from ID 20002, code 3,4 or 5 from ID 20126 and code 11 from ID 20544 as case. In order to obtain a comprehensive and accurate control group, we strictly conducted the control group threshold by Davis et al. research[[2](#_ENREF_2)], which based on Patient Health Questionnaire (PHQ-9) and another strict criterion based on composite international diagnostic interview short-form (CIDI-SF)[[3](#_ENREF_3), [4](#_ENREF_4)].

For the control of the depression, after excluding the depression defined in our study and depression single episode defined in Davis et al. research[[2](#_ENREF_2)], we chose the participants who did not endorse depression or screen positive on PHQ or CIDI. More precisely, participants whose PHQ score ≤5 and did not have core symptoms were selected. PHQ-9[[3](#_ENREF_3)] is a classification algorithm with a total score (0-27) used to screen for and measure depression severity, focusing on nine depressive symptoms and signs (as detailed below：Little interest or pleasure in doing things 20514, Feeling down, depressed, or hopeless 20510, Trouble sleeping 20517, Feeling tired 20519, Poor appetite or overeating 20511, Feeling bad about yourself 20507, Trouble concentrating 20508, Moving or speaking slowly or fidgety or restless 20518, Thoughts that you would be better off dead 20513). In order to meet the 0-3 score for each item of PHQ, the 9 symptom scores (1-4) of our team UK were all reduced by 1 point, which was then added up. According to CIDI[[4](#_ENREF_4)], core symptoms of depression were ID 20446 and ID 20441 in UK Biobank, we chose the participants who response “NO” to the question “Have you ever had a time in your life when you felt sad, blue, or depressed for two weeks or more in a row?” or “Have you ever had a time in your life lasting two weeks or more when you lost interest in most things like hobbies, work, or activities that usually give you pleasure?” as non-depressed patients.

**Questionnaire wording and format**

Introduction

Section A: present and past depression.

Section B: generalized anxiety disorder.

| **Section A: present and past depression** | | | | |
| --- | --- | --- | --- | --- |
| **PHQ score:** | | | | |
| 1. **20514** 2. **20510** 3. **20534** 4. **20519** 5. **20511** 6. **20507** 7. **20508** 8. **20518** 9. **20513** | | Over the last 2 weeks, how often have you been bothered by any of the following problems?  a. Little interest or pleasure in doing things  b. Feeling down, depressed, or hopeless  c. Trouble falling or staying asleep, or  sleeping too much  d. Feeling tired or having little energy  e. Poor appetite or overeating  f. Feeling bad about yourself or that you  are a failure or have let yourself or your  family down  g. Trouble concentrating on things, such  as reading the newspaper or watching  television  h. Moving or speaking so slowly that other  people could have noticed? Or the opposite — being so fidgety or restless that you have been moving around a lot more than usual  i. Thoughts that you would be better off  dead or of hurting yourself in some way | [Select one from the following for each of the statements]  - 01 Not at all  - 02 Several days  - 03 More than half the days  - 04 Nearly every day  - DA Prefer not to answer | |
| **Section B: generalized anxiety disorder** | | | | |
| **GAD-7** | | | | |
| 1. **20506** 2. **20509** 3. **20520** 4. **20515** 5. **20516** 6. **20505** 7. **20512** | Over the last 2 weeks, how often have you been bothered by any of the following problems?  a) Feeling nervous, anxious or on edge  b) Not being able to stop or control worrying  c) Worrying too much about different things  d) Trouble relaxing  e) Being so restless that it is hard to sit still  f) Becoming easily annoyed or irritable  g) Feeling afraid as if something awful might happen  [7 questions on one screen in grid] | | | [Select one from the following for each of the statements]  - 01 Not at all  - 02 Several days  - 03 More than half the days  - 04 Nearly every day  - DA Prefer not to answer |

**Reference**

[1] Spitzer RL, Kroenke K, Williams JB, Löwe B. A brief measure for assessing generalized anxiety disorder: the GAD-7. Archives of internal medicine. 2006;166:1092-7.

[2] Davis KAS, Cullen B, Adams M, Brailean A, Breen G, Coleman JRI, et al. Indicators of mental disorders in UK Biobank—A comparison of approaches. International Journal of Methods in Psychiatric Research. 2019;28:e1796.

[3] Kroenke K, Spitzer RL, Williams JBW, L?we B. The Patient Health Questionnaire Somatic, Anxiety, and Depressive Symptom Scales: a systematic review.32:345-59.

[4] Kessler RC, Andrews G, Mroczek D, Ustun B, Wittchen Hl. The World Health Organization Composite International Diagnostic Interview short‐form (CIDI㏒F). International Journal of Methods in Psychiatric Research. 1998.
