# Supplementary material for: Genome-wide association studies in non-anxiety individuals identified novel risk loci for depression
Source: Eur Psychiatry. 2022 Jun 22;65(1):e38. doi: 10.1192/j.eurpsy.2022.32 (PMC9353885; doi:10.1192/j.eurpsy.2022.32)
Supplement: Supplementary file 1 [file S0924933822000323.zip › S0924933822000323sup003.docx]

**Supplementary file 2. Descriptive characteristics of anxiety score < 5 participants**

| **Participants with anxiety score < 5** | **No.** | **Mean ± SD** | **Range** |
| --- | --- | --- | --- |
| **Depression score samples** |  |  |  |
| Age, years | 99,178 | 56.61 ± 7.52 | 39 - 72 |
| Sex |  |  |  |
| Male | 53,157 |  |  |
| Female | 46,021 |  |  |
| TDI | 99,069 | -1.96 ± 2.68 | -6.26 - 10.46 |
| Alcohol use frequency/week | 83,912 | 10.26 ± 9.07 | 0 - 256 |
| Smoking frequency/day | 84,289 | 5.50 ± 9.79 | 0 - 140 |
| **Self-reported depression** |  |  |  |
| ***Case*** |  |  |  |
| Age, years | 59,334 | 57.22 ± 7.46 | 40 - 72 |
| Sex |  |  |  |
| Male | 27,900 |  |  |
| Female | 31,434 |  |  |
| TDI | 59,280 | -2.12 ± 2.58 | -6.26 - 9.40 |
| Alcohol use frequency/week | 51,331 | 10.51 ± 9.11 | 0.00 - 256 |
| Smoking frequency/day | 50,327 | 5.00 ± 9.44 | 0 - 140 |
| ***Control*** |  |  |  |
| Age, years | 19,805 | 55.77 ± 7.40 | 40 - 70 |
| Sex |  |  |  |
| Male | 13,089 |  |  |
| Female | 6,716 |  |  |
| TDI | 19,775 | -1.64 ± 2.82 | -6.26 - 9.14 |
| Alcohol use frequency/week | 16,220 | 9.77 ± 9.11 | 0 - 118 |
| Smoking frequency/day | 16,868 | 6.63 ± 10.38 | 0 - 100 |

TDI, Townsend deprivation index.
