# Supplementary material for: Genome-wide association studies in non-anxiety individuals identified novel risk loci for depression
Source: Eur Psychiatry. 2022 Jun 22;65(1):e38. doi: 10.1192/j.eurpsy.2022.32 (PMC9353885; doi:10.1192/j.eurpsy.2022.32)
Supplement: Supplementary file 1 [file S0924933822000323.zip › S0924933822000323sup004.docx]

**Supplementary file 3. Descriptive characteristics of non-self-reported anxiety participants**

| **Participants with non-self-reported anxiety** | **No.** | **Mean ± SD** | **Range** |
| --- | --- | --- | --- |
| **Depression score samples** |  |  |  |
| Age, years | 86,503 | 56.71 ± 7.55 | 39 - 72 |
| Sex |  |  |  |
| Male | 45,066 |  |  |
| Female | 41,437 |  |  |
| TDI | 86,412 | -1.98 ± 2.66 | -6.26 - 10.46 |
| Alcohol use frequency/week | 73,598 | 10.31 ± 9.09 | 0 - 256 |
| Smoking frequency/day | 73,521 | 5.39 ± 9.74 | 0 - 140 |
| **Self-reported depression** |  |  |  |
| ***Case*** |  |  |  |
| Age, years | 56,603 | 57.21 ± 7.48 | 40 - 72 |
| Sex |  |  |  |
| Male | 26,383 |  |  |
| Female | 30,220 |  |  |
| TDI | 56,552 | -2.12 ± 2.58 | -6.26 - 9.40 |
| Alcohol use frequency/week | 49,009 | 10.52 ± 9.14 | 0 - 256 |
| Smoking frequency/day | 48,019 | 4.99 ± 9.44 | 0 - 140 |
| ***Control*** |  |  |  |
| Age, years | 13,123 | 55.90 ± 7.45 | 40 - 70 |
| Sex |  |  |  |
| Male | 8,669 |  |  |
| Female | 4,454 |  |  |
| TDI | 13,102 | -1.66 ± 2.82 | -6.26 - 8.94 |
| Alcohol use frequency/week | 10,788 | 9.71 ± 8.98 | 0 - 118 |
| Smoking frequency/day | 11,168 | 6.52 ± 10.36 | 0 - 100 |

TDI, Townsend deprivation index.
