# Supplementary material for: Genome-wide association studies in non-anxiety individuals identified novel risk loci for depression
Source: Eur Psychiatry. 2022 Jun 22;65(1):e38. doi: 10.1192/j.eurpsy.2022.32 (PMC9353885; doi:10.1192/j.eurpsy.2022.32)
Supplement: Supplementary file 1 [file S0924933822000323.zip › S0924933822000323sup005.docx]

**Supplementary file 4. Genome-wide candidate loci in non-anxiety depression showing the candidate genes**

| GWAS | Gene | SNP | REF | ALT | OR | *P* |
| --- | --- | --- | --- | --- | --- | --- |
| Self-reported depression 1 | CFAP61 | rs6046722 | C | A | 1.09 | 2.52 × 10^−8^ |
|  | CFAP61 | rs2424298 | T | C | 1.09 | 3.58 × 10^−6^ |
| Depression score 1 | PIEZO2 | rs139702470 | A | G | 0.29 | 1.54 × 10^−8^ |
| Self-reported depression 2 | CFAP61 | rs4814959 | A | G | 1.08 | 8.53 × 10^−6^ |
|  | CFAP61 | rs6046722 | C | A | 1.1 | 1.14 × 10^−7^ |
|  | CFAP61 | rs79469779 | T | C | 1.11 | 6.94 × 10^−6^ |
| Depression score 2 | PIEZO2 | rs139702470 | A | G | 0.29 | 3.66 × 10^−8^ |

Note: GWAS, genome wide association study. Self-reported depression 1, GWAS of self-reported depression conducted in individuals with anxiety score < 5. Depression score 1, GWAS of depression score conducted in individuals with anxiety score < 5. Self-reported depression 2, GWAS of self-reported depression conducted in individuals with non-self-reported anxiety. Depression score 2, GWAS of depression score conducted in individuals with non-self-reported anxiety.
