# Supplementary material for: Genome-wide association studies in non-anxiety individuals identified novel risk loci for depression
Source: Eur Psychiatry. 2022 Jun 22;65(1):e38. doi: 10.1192/j.eurpsy.2022.32 (PMC9353885; doi:10.1192/j.eurpsy.2022.32)
Supplement: Supplementary file 1 [file S0924933822000323.zip › S0924933822000323sup006.docx]

**Supplementary file 5. Replication of primary analysis results in *CFAP61* region**

| SNP | Location | REF | ALT | Replication GWAS 1 | | | Replication GWAS 2 | | |
| --- | --- | --- | --- | --- | --- | --- | --- | --- | --- |
|  |  |  |  | OR | SE | *P* | OR | SE | *P* |
| rs1040582 | 20198644 | T | C | 0.97 | 0.01 | 0.02 | 0.96 | 0.01 | 2.71 × 10^−3^ |
| rs13038510 | 20080830 | T | C | 1.03 | 0.01 | 0.02 | 1.04 | 0.01 | 9.83× 10^−3^ |
| rs17310719 | 20277062 | A | G | 0.97 | 0.02 | 0.05 | 0.95 | 0.01 | 1.45× 10^−2^ |
| rs6035550 | 20107627 | A | T | 0.98 | 0.01 | 0.03 | 0.97 | 0.01 | 2.97× 10^−3^ |
| rs6075614 | 20098716 | A | G | 0.98 | 0.01 | 0.03 | 0.97 | 0.01 | 3.02× 10^−2^ |
| rs6081881 | 20120290 | T | C | 0.97 | 0.01 | 0.02 | 0.97 | 0.01 | 2.12× 10^−2^ |
| rs6081920 | 20207860 | T | C | 0.97 | 0.01 | 0.02 | 0.96 | 0.01 | 2.57× 10^−3^ |
| rs6081921 | 20209860 | A | G | 0.97 | 0.01 | 0.02 | 0.96 | 0.01 | 2.48× 10^−3^ |
| rs75454869 | 20213030 | A | G | 0.97 | 0.01 | 0.03 | 0.96 | 0.01 | 2.81× 10^−3^ |
