# Supplementary material for: Genome-wide association studies in non-anxiety individuals identified novel risk loci for depression
Source: Eur Psychiatry. 2022 Jun 22;65(1):e38. doi: 10.1192/j.eurpsy.2022.32 (PMC9353885; doi:10.1192/j.eurpsy.2022.32)
Supplement: Supplementary file 1 [file S0924933822000323.zip › S0924933822000323sup007.docx]

**Supplementary file 6. Replication of primary analysis results in *PIEZO2* region**

| SNP | Location | REF | ALT | Replication GWAS 1 | | | Replication GWAS 2 | | |
| --- | --- | --- | --- | --- | --- | --- | --- | --- | --- |
|  |  |  |  | OR | SE | *P* | OR | SE | *P* |
| rs11661122 | 10945734 | T | G | 0.97 | 0.01 | 8.16 × 10^−3^ | 0.97 | 0.01 | 8.08 × 10^−3^ |
| rs11664237 | 10936838 | T | C | 0.97 | 0.01 | 8.58 × 10^−4^ | 0.97 | < 0.01 | 3.46 × 10^−3^ |
| rs12608436 | 10797858 | A | G | 1.02 | 0.01 | 4.62 × 10^−2^ | 1.02 | < 0.01 | 3.76 × 10^−2^ |
| rs1918683 | 10792623 | T | C | 0.98 | 0.01 | 8.43 × 10^−3^ | 0.97 | 0.01 | 4.75 × 10^−3^ |
| rs2865133 | 10811337 | A | G | 1.02 | 0.01 | 2.09 × 10^−2^ | 1.03 | 0.01 | 7.92 × 10^−3^ |
| rs4411565 | 10809063 | A | T | 1.03 | 0.01 | 8.42 × 10^−3^ | 1.03 | 0.01 | 3.89 × 10^−3^ |
| rs58703063 | 10785880 | T | C | 1.02 | 0.01 | 3.07 × 10^−2^ | 1.02 | < 0.01 | 2.40 × 10^−2^ |
| rs6505593 | 10791750 | A | G | 0.98 | 0.01 | 9.50 × 10^−3^ | 0.97 | 0.01 | 4.64 × 10^−3^ |
| rs6505599 | 10806869 | T | C | 1.02 | 0.01 | 9.99 × 10^−3^ | 1.04 | 0.01 | 3.05 × 10^−3^ |
| rs7227426 | 10802410 | T | C | 0.98 | 0.01 | 1.45 × 10^−2^ | 0.97 | 0.01 | 8.49 × 10^−3^ |
| rs72874285 | 11098960 | T | C | 0.97 | 0.01 | 1.04 × 10^−2^ | 0.97 | 0.01 | 1.50 × 10^−2^ |
| rs9303717 | 10793275 | T | C | 0.98 | 0.01 | 1.06 × 10^−2^ | 0.97 | 0.01 | 5.20 × 10^−3^ |
| rs9945135 | 10806165 | T | C | 1.02 | 0.01 | 1.02 × 10^−2^ | 1.04 | 0.01 | 3.02 × 10^−3^ |
| rs9973179 | 10809214 | A | G | 1.02 | 0.01 | 1.06 × 10^−2^ | 1.03 | 0.01 | 3.62 × 10^−3^ |
